# Nearest-Neighbor Projected Distance Regression for Epistasis Detection in GWAS With Population Structure Correction

**DOI:** 10.3389/fgene.2020.00784

**Published:** 2020-07-22

**Authors:** Marziyeh Arabnejad, Courtney G. Montgomery, Patrick M. Gaffney, Brett A. McKinney

**Affiliations:** ^1^Tandy School of Computer Science, University of Tulsa, Tulsa, OK, United States; ^2^Arthritis and Clinical Immunology Research Program, Oklahoma Medical Research Foundation, Oklahoma City, OK, United States; ^3^Department of Mathematics, University of Tulsa, Tulsa, OK, United States

**Keywords:** epistasis, feature selection, GWAS, machine learning, nearest-neighbors

## Abstract

Nearest-neighbor Projected-Distance Regression (NPDR) is a feature selection technique that uses nearest-neighbors in high dimensional data to detect complex multivariate effects including epistasis. NPDR uses a regression formalism that allows statistical significance testing and efficient control for multiple testing. In addition, the regression formalism provides a mechanism for NPDR to adjust for population structure, which we apply to a GWAS of systemic lupus erythematosus (SLE). We also test NPDR on benchmark simulated genetic variant data with epistatic effects, main effects, imbalanced data for case-control design and continuous outcomes. NPDR identifies potential interactions in an epistasis network that influences the SLE disorder.

## Introduction

An important challenge for machine learning in GWAS is to perform computationally efficient screening for variants involved in complex genetic models, including epistatic effects. The identification of interactions in GWAS may lead to an increased understanding of pathogenic mechanisms and potential therapeutic targets, but low minor allele frequencies and the curse of dimensionality make interaction detection difficult. Machine learning methods also face the challenge of identifying statistical thresholds that limit false discoveries and handling the intricacies of biomedical studies such as covariates and population structure.

Recently we developed a flexible nearest-neighbor-based machine learning feature selection method called Nearest-neighbor Projected Distance Regression (NPDR) to address these challenges (Le et al., 2020). NPDR integrates a regression formalism to allow statistical significance testing with projected nearest-neighbor machine learning to enable detection of complex multivariate models in high dimensional data. The projection of nearest neighbors from high dimensions onto single feature dimensions allows NPDR to detect features involved in complex patterns with other features in high-dimensional data that influence phenotypic variance. The regression formalism of NPDR maintains the ability to detect interactions while providing a statistical basis for feature selection thresholding and control of false discoveries due to multiple hypothesis testing.

In the current study, we demonstrate the capabilities of the NPDR framework to detect variants involved in complex genetic models and to adjust for population structure. We compare the performance of NPDR with random forest and univariate analysis on a panel of benchmark simulated genetic variant data described by Urbanowicz et al. (2018). We analyze data with multivariate main effects and multiple epistatic effects and outcomes with balanced and imbalanced cases-control ratios as well as continuous variation. Consistent with our previous studies (McKinney et al., 2009; Le et al., 2020), we show that random forest is able to detect interactions when the number of predictors is small but its power diminishes with the dimensionality of the data. NPDR is less susceptible to the curse of dimensionality as we show it is able to detect interactions with statistical significance in both low and high dimensional contexts.

In addition to adjustment for multiple testing, NPDR enables the adjustment for covariates such as sex, age, or population structure – due to population stratification or cryptic relatedness. Population structure leads to linkage disequilibrium (LD) and this deviation from independence may increase false associations (McCarthy et al., 2008; Chen et al., 2016). The confounding effect of population structure may be exacerbated for complex models involving interactions between variants. Covariate adjustment is challenging for many machine learning methods that have the flexibility of being model free (Le et al., 2020). NPDR is model free in its use of nearest neighbors for detecting interactions, but it includes a statistical model for the projected distance for each feature. This generalized linear model (GLM) of projected distances then allows for the inclusion of projected distance covariates such as principal components (PCs).

Systemic lupus erythematosus (SLE) is an autoimmune inflammatory disease characterized by antinuclear autoanti bodies, complement and interferon activation, and tissue destruction. It predominantly affects women. Numerous immune-related genes and genes with other functions have been shown to predispose to SLE (Harley et al., 2008; Gregersen and Olsson, 2009), but there is a need to identify other genomic factors that may be interacting with each other as pairs or in a higher-order network to influence the development of this complex disease (Davis et al., 2013; Tyler et al., 2019). We use NPDR to enrich for interactions in the systemic lupus erythematosus genetics (SLEGEN) GWAS, which consists of females of European ancestry (720 SLE and the 2,337 controls) (Harley et al., 2008). Although the SLEGEN data is a homogeneous sample, we demonstrate the ability of NPDR to adjust for possible cryptic relatedness by including PCs as covariates. Identifying additional interacting variants may lead to a better understanding of the pathways affecting SLE.

## Materials and Methods

### Nearest-Neighbor Projected-Distance Regression

Relief-based methods are known for their ability to identify interactions with computational efficiency but generally do not account for statistical significance of the attributes that may lead to high misclassification rate. In order to control false discoveries and adjust for covariates, we developed NPDR to use the GLM to perform regression between nearest-neighbor pair distances projected onto each predictor dimension (Le et al., 2020). We define the NPDR neighborhood set 𝒩 of ordered pair indices of subjects as follows.

In NPDR, instance (e.g., subject) *i* is a point in *p* attribute (e.g., variant) dimensions, and the topological neighborhood of *i* is labeled by *N*_*i*_. This neighborhood is a set of other instances trained on the dataset *X*^*m*×*p*^ of *m* instances and *p* attributes and depends on the type of Relief neighborhood method (e.g., fixed-*k* or adaptive radius) and the type of metric (e.g., Manhattan or Euclidean). If instance *j* is in the neighborhood of *i*(*j* ∈ *N*_*i*_), then the ordered pair is in the overall neighborhood ((*i*, *j*) ∈ 𝒩) for the projected-distance regression analysis. The ordered pairs constituting the overall neighborhood can then be represented as nested sets:

$$𝒩={\left\{{\left\{\left(i,j\right)\right\}}_{i=1}^{m}\right\}}_{\left\{j\neq i:j\in N_{i}\right\}}.$$

The cardinality of the set {*j* ≠ *i* : *j* ∈ *N*_*i*_} is *k*_*i*_, the number of nearest neighbors for subject *i*. In the analyses in the current study, we use an adaptive *k* for hits and misses, *k* = 0.154 (m-1), that has shown good balance between detecting main effects and interaction effects (Le et al., 2019, 2020).

We compute the distance between two instances *i* and *j* in the space of the set A of all attributes with an *L*_*q*_ metric

$$D_{i\,j}^{\left(q\right)}={\left(\sum_{a\in A}{\left|d_{i\,j}\,\left(a\right)\right|}^{q}\right)}^{1/q},$$

where |*A*| = *p* is the number of attributes in the dataset. We use *q* = 1 (Manhattan) in this study. The projected difference or diff function [*d*_*ij*_(*a*)] between two instances *i* and *j* onto a SNP is of critical importance to the NDPR algorithm and can be computed by various difference functions. The standard difference used by Relief-based algorithms for categorical variables is a binary mismatch. For SNPs, this genotype mismatch (GM) is a 0 or 1 difference between two individuals (*R*_*i*_, *R*_*j*_) for a SNP, *a*, based on the individuals’ genotypes for this SNP. Specifically, the diff function is

$$\begin{array}{c} d_{i\,j}^{G\,M}\,\left(a\right)={\text{diff}}_{G\,M}\,\left(a,R_{i},R_{j}\right)\\ \;=\left\{\begin{array}{cc} 0, & \text{genotype}\,\left(a,\,R_{i}\right)=\text{genotype}\,\left(a,R_{j}\right)\\ 1, & \text{otherwise} \end{array}\right\} \end{array}$$

where genotype (*a*, *R*_*i*_) is the genotype for individual *R*_*i*_ for SNP *a*. In other words, two individuals have zero diff if they have identical genotypes and they have unit diff if they have different genotypes.

A potential drawback of GM is that it is not sensitive to heterozygous genotype differences when computing the diff. The following allele mismatch (AM) diff accounts for the difference in the number of alleles for a SNP when computing the distance between two individuals (Arabnejad et al., 2018). The AM difference of two individuals can be calculated by the following formula:

$$\begin{array}{c} d_{i\,j}^{A\,M}\,\left(a\right)={\text{diff}}_{A\,M}\,\,\left(g_{\nu },R_{i},R_{j}\right)\\ \;=\frac{1}{2}\times \left|\text{genotype}\,\left(a,\,R_{i}\right)-\text{genotype}\,\left(a,\,R_{j}\right)\right| \end{array}$$

where genotype(*a*, *R*_i_) is the genotype encoding for number of copies of the reference allele forSNP *a* for individual *i*. In other words, the value of genotype(*a*, *R*_i_) is the number of minor alleles in the genotype: 0, 1, or 2. Then the return value of $d_{i\,j}^{A\,M}\,\left(a\right)$ is 0, 0.5, or 1 when the two individual have 2, 1, or 0 alleles in common, respectively. Other projected differences and metrics are described by Arabnejad et al. (2018).

Nearest-neighbor Projected-Distance Regression uses the GLM to perform regression between nearest neighbors. For each attribute, the NPDR model fits a GLM to the attribute’s projected distances between all pairs of nearest neighbors. The regression coefficients are calculated to minimize the least-squares error. For case-control phenotypes, $p_{i\,j}^{\text{miss}}$ is the probability that subjects i and j are in the opposite phenotype class (misses) versus the same class (hits). We model this probability from the projected differences for SNP *a* with logit function:

$$\text{logit}\,\left(p_{i\,j}^{m\,i\,s\,s}\right)=\beta_{0}+\beta_{a}\,d_{i\,j}\,\left(a\right)+\varepsilon_{i\,j}.$$

The NPDR test statistic for attribute *a* is the β_*a*_ estimate with one-sided hypotheses:

$$\begin{array}{c} H_{0}:\beta_{a}<0.\\ H_{0}:\beta_{a}\geq 0. \end{array}$$

The quantity *e*^β_*a*_^ is the predicted change in odds of neighbors being in opposite classes when the difference of the attribute *a* changes by one unit. For a continuous outcome (quantitative trait), NPDR uses linear regression of the numerical difference $d_{i\,j}^{\text{num}}\,\left(y\right)$ of the outcome *y* between neighbors:

$$d_{i\,j}^{\text{num}}\,\left(y\right)=\beta_{0}+\beta_{a}\,d_{i\,j}\,\left(a\right)+\varepsilon_{i\,j}$$

and feature importance and significance are again determined from the coefficient β_*a*_.

False-positive associations can arise in GWAS due to population stratification or cryptic relatedness. A standard approach to correct for population structure is to include PCs in the regression model to account for the genetic background. Many machine learning feature selection algorithms have limited ability to adjust for population structure or other potentially confounding covariates. However, the NPDR formalism can adjust for multiple covariates by including projected differences $d_{i\,j}^{\text{num}}\,\left({\vec{y}}_{\text{covs}}\right)$ for each covariate attribute in the regression model. The covariate adjusted model then becomes,

$$\text{logit}\,\left(p_{i\,j}^{\text{miss}}\right)=\beta_{0}+\beta_{a}\,d_{i\,j}\,\left(a\right)+{\vec{\beta }}_{\text{covs}}^{T}\,d_{i\,j}\,\left({\vec{y}}_{\text{covs}}\right)+\varepsilon_{i\,j}$$

where the covariate coefficient vector for 10 PCs is,

$${\vec{\beta }}_{\text{covs}}^{T}=\left(\beta_{P\,C_{1}},\beta_{P\,C_{2}},\ldots ,\beta_{P\,C_{10}}\right)$$

and again $p_{i\,j}^{\text{miss}}$ is the probability of neighbors having a different phenotype. Neighbors are still determined in the attribute (variant) space, but we add additional covariate diffs to the NDPR regression model.

### Simulated Data

We compare methods using existing simulated data from the epistasis benchmark described by Urbanowicz et al. (2018) and available at https://github.com/EpistasisLab/rebate-benchmark/. For simplicity, many of the benchmark simulations use 20 total variants, but we also compare performance for multiple replicate simulations with 10,000 total variants to demonstrate computational feasibility and the effect of higher dimensionality (Table 1 summary of datasets). For case-control data, we use data with 1,600 balanced instances (800 cases and 800 controls) and one imbalanced scenario with (60% cases). Datasets have a heritability effect size of 0.4, minor allele frequency of 0.2 and include models with 2–4 functional variants and models with additive main effects and epistatic effects. We also use a dataset with a pair of interacting variants that influence a continuous endpoint.

**TABLE 1:** Properties of simulated data from epistasis benchmarking repository (https://github.com/EpistasisLab/rebate-benchmark/).

Dataset	Predictive features (influence ratio)	Total features	Heritability	MAF	Instances (case/ctl)
Main effect pair	2 (50:50)	20	0.4	0.2	800/800
4-main effect	4 (25:25:25:25)	20	0.4	0.2	800/800
4 interactions	4 (2 interacting pairs)	20	0.4	0.2	800/800
Interacting pair	2	20	0.05	0.2	800/800
Continuous outcome interactions	2	20	0.4	0.2	1,600
Imbalanced outcome interactions	2	20	0.4	0.2	960/640
10,000 variants	2	10,000	0.4	0.2	800/800

*All scenarios are case-control except one continuous outcome dataset. All scenarios have 20 features except for one with 10,000 features.*

### Real GWAS

We apply NPDR to a study of females with European ancestry with genotyping data for 317,503 SNPs for 720 SLE subjects and 2,337 controls from the SLEGEN consortium (Harley et al., 2008). All women with SLE satisfied the revised criteria for classification of SLE from the American College of Rheumatology. The study sample consisted of 730 unrelated women with SLE and 475 controls from SLEGEN. Additional “out-of-study” European ancestry female controls were added from Illumina’s iControlDB. The majority of iControlDB are from the Robert S. Boas Center for Genomics and Human Genetics at the Feinstein Institute for Medical Research. We reduced the data dimensionality using LD pruning with a correlation threshold of 0.5, minor allele frequency threshold of 0.01, Hardy-Weinberg Expectations (HWE) in controls *P* > 0.01, and HWE in cases *P* > 0.0001. LD pruning helps remove redundant features, where a SNP from a pair in high LD is removed from the data (Calus and Vandenplas, 2018). Initial filtering reduced the number of SNPs to 184,170. Due to computer memory constraints of the current implementation of NPDR, we further filtered to 10,000 SNPs based on univariate association. This filtering risks removing some interaction effects but should capture a considerable amount of variation in the data. Future implementations of NPDR will improve memory efficiency and incorporate additional variants. An advantage of NPDR is the ability to incorporate covariates into feature selection. We used the top 10 PCs from the variance-standardized relationship matrix. We mapped the top SNPs found by NPDR to Ensembl gene IDs based on proximity. If the SNP is not in an intron or exon of a gene, the algorithm computes the distance of the variant to the nearest two genes and the SNP is mapped to the gene symbol of the closest gene.

## Results

### Simulation Analysis

**FIGURE 1:**
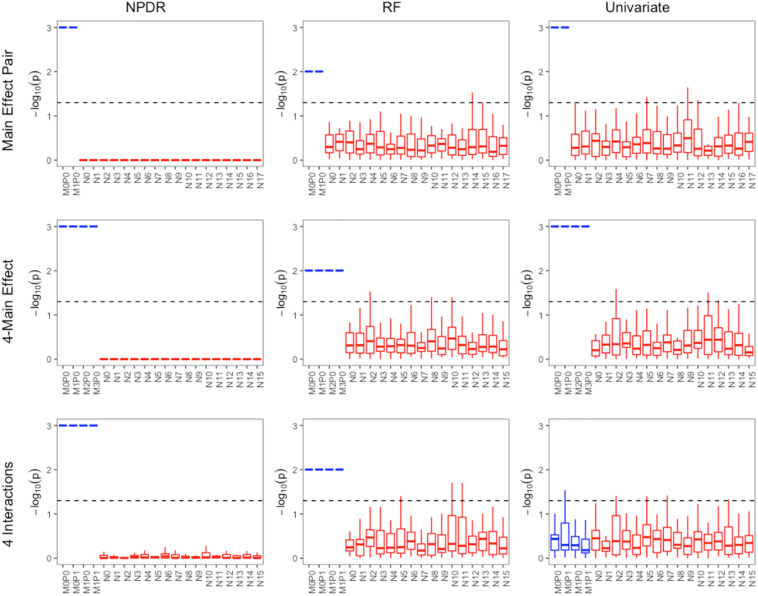
Performance of three feature selection algorithms (from left to right): NPDR, random forest (RF) using permutation *P*-value, and univariate regression for three simulation models from https://github.com/EpistasisLab/rebate-benchmark/. Simulated models include (from top to bottom) main effect of two variants, main effect of four variants, and heterogeneous interaction of two interacting pairs. The –log(adjusted *P*-value) is plotted for the 20 variants in each dataset for 30 replicate simulations. The functional variable names (blue) begin with letter M, and the background variable names (red) start with letter N. Datasets have 1,600 samples (800 cases and 800 controls). Additional dataset details are given in Table 1. The dashed line represents Bonferroni adjusted *P*-value of 0.05.

**FIGURE 2:**
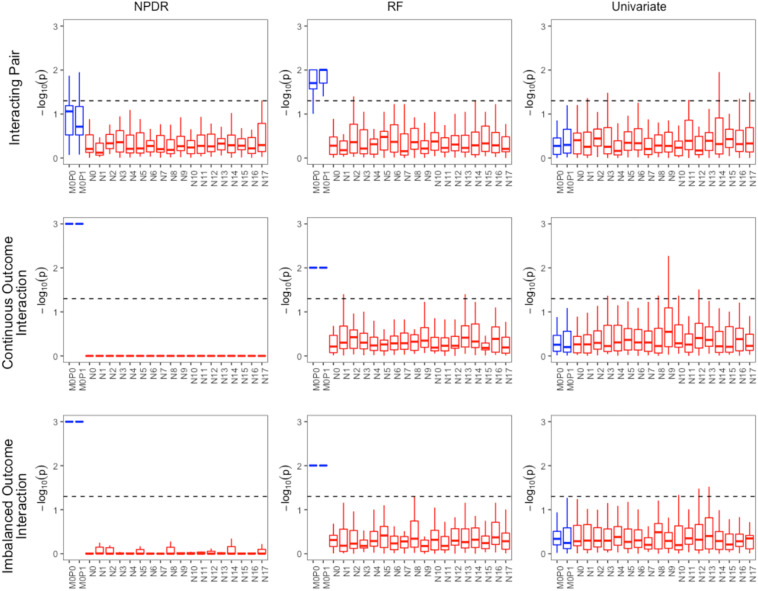
Performance of three feature selection algorithms (from left to right): NPDR, random forest (RF) using permutation *P*-value, and univariate regression for three simulation models from https://github.com/EpistasisLab/rebate-benchmark/. Simulated models include (from top to bottom) an interacting pair, an interacting pair with a continuous outcome, and imbalanced case-control (60%). The –log(adjusted *P*-value) is plotted for the 20 variants in each dataset for 30 replicate simulations. The functional variable names (blue) begin with letter M, and the background variable names (red) start with letter N. Datasets have 1,600 samples (800 cases and 800 controls). Additional dataset details are given in Table 1. The dashed line represents Bonferroni adjusted *P*-value of 0.05.

**FIGURE 3:**
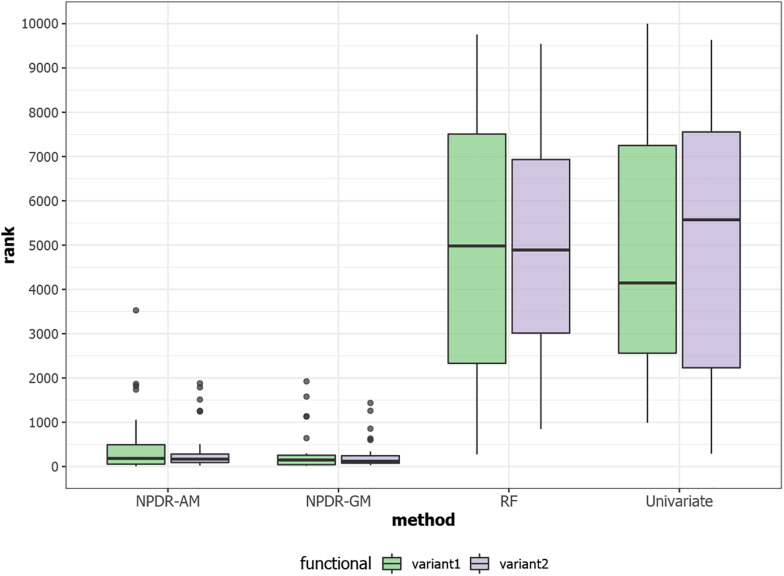
Rank (lower is better) of the two functional interacting variants [variant 1 (green) and variant 2 (purple)] in simulated datasets with 10,000 total variants from https://github.com/EpistasisLab/rebate-benchmark/. The ranks of the two functional variables are averaged over 30 replicate simulations. NPDR *P*-value ranking is performed for allele mismatch (AM) and genotype mismatch (GM) projected distance metrics. Random forest ranking is performed by permutation importance score (RF) and univariate uses the regression coefficient *P*-value. The 10,000 simulated variants have average minor allele frequency 0.2 and heritability 0.4, and datasets have 800 cases and 800 controls.

For low-dimensional simulations with only 20 total attributes (Figures 1, 2), both NPDR with AM metric and random forest rank the functionally interacting attributes at the top for all simulation scenarios. All methods detect the main effects (Figure 1), but as expected the univariate analysis cannot detect interaction effects. For low dimensional datasets (20 attributes), random forest is able to exhaustively sample all attributes and find a tree with the interacting attributes. When the total number of attributes increases from 20 to 10,000 (Figure 3), random forest is unable to detect the functional interactions with average ranks near random (5,000). In this case of 10,000 attributes, the random forest ranking is very similar to a univariate ranking, while NPDR has good rankings for interacting attributes using either the GM or AM projected difference metrics (Figure 3). This is consistent with our previous results that random forest is unable to detect interactions beyond random chance in high dimensional data, whereas Relief-based methods are less affected by the curse of dimensionality (McKinney et al., 2009). NPDR also performs well for multiple pairwise interactions (Figure 1), interactions for a continuous outcome (Figure 2, middle row) and imbalanced case-control data (Figure 2, bottom row).

### SLE GWAS

**FIGURE 4:**
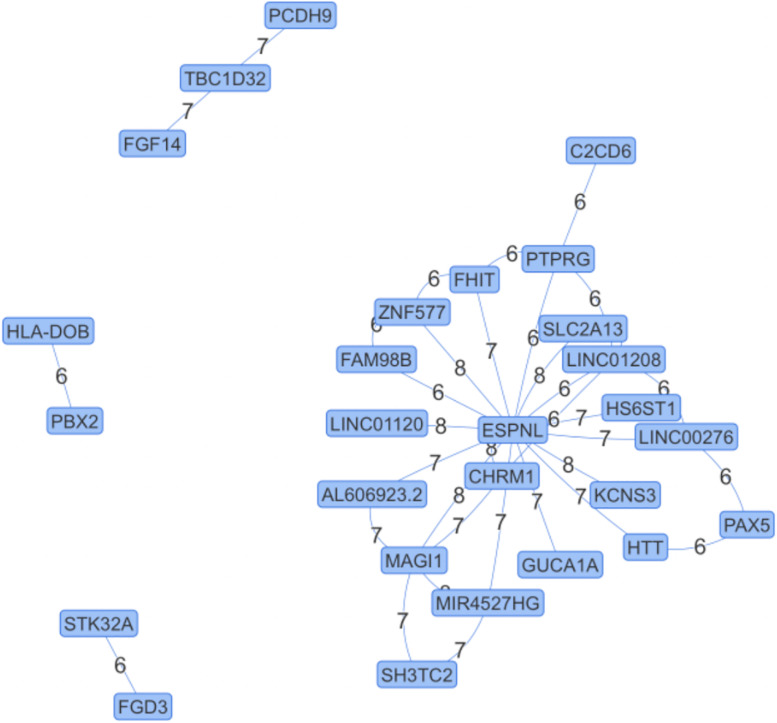
Epistasis Network for Lupus GWAS. Edges are significant pairwise interactions with adjusted *P*-value < 10^–6^ between variants in genes computed after filtering. The edge weights are the magnitude of the statistical interaction between SNPs calculated by –log_10_ (adjusted *P*-value). The espin-like (ESPNL) gene is an epistasis hub with 15 edges. The interaction on the left (HLA-DOB and PBX2) is between genes in the MHC II region.

We apply NPDR to the SLEGEN data, which is a real GWAS composed of females with SLE and healthy controls. Although the study is composed of European ancestry individuals, we include 10 PCs as covariates in NPDR to account for possible cryptic relatedness. Following filtering we create a network from significant (adjusted *P*-value < 1e-6) pairwise interactions (Figure 4). This edge significance threshold results in 35 edges (Table 2) in the epistasis network. The espin-like (ESPNL) gene is a hub of the network, involved in 15 of the 35 significant interactions. The particular interacting SNP (rs10210979) in ESPNL is an intron variant on chromosome 2. Although ESPNL is involved in hearing, it is ubiquitously expressed and some of its interactions may indicate novel function for the immune system. In addition to this hub gene, there is an interesting interaction between HLA-DOB and PBX2 (Pre-B-cell leukemia homeobox 2). Both of these genes are located within the major histocompatibility complex (MHC) class II region on chromosome 6, and HLA-DOB is a beta chain of the MHC class II molecule.

**TABLE 2:** Logistic regression interaction statistics for pairs of variants in genes in the epistasis network (Figure 4) for SLE GWAS with adjusted *P*-values < 10^–6^.

SNP 1	Gene 1	SNP 2	Gene 2	*P*-value	Adjusted *P*-value
rs10210979	ESPNL	rs2067477	CHRM1	5.149E-12	3.51E-09
rs10210979	ESPNL	rs11564281	SLC2A13	1.055E-11	3.51E-09
rs10210979	ESPNL	rs4832401	KCNS3	1.243E-11	3.51E-09
rs10210979	ESPNL	rs7573771	LINC01120	4.106E-11	8.70E-09
rs10210979	ESPNL	rs9814172	MAGI1	1.585E-10	2.69E-08
rs10210979	ESPNL	rs9807842	ZNF577	2.431E-10	3.08E-08
rs9814172	MAGI1	rs6507759	MIR4527HG	2.539E-10	3.08E-08
rs10210979	ESPNL	rs7694687	HTT	4.235E-10	4.16E-08
rs9814172	MAGI1	rs2067477	CHRM1	4.415E-10	4.16E-08
rs10210979	ESPNL	rs1446540	LINC00276	9.587E-10	8.13E-08
rs10210979	ESPNL	rs7762152	GUCA1A	1.189E-09	9.17E-08
rs9814172	MAGI1	rs17653341	SH3TC2	1.568E-09	1.05E-07
rs10210979	ESPNL	rs9311738	FHIT	1.606E-09	1.05E-07
rs10210979	ESPNL	rs6507759	MIR4527HG	2.278E-09	1.38E-07
rs11920836	LINC01208	rs11564281	SLC2A13	2.638E-09	1.44E-07
rs17083190	TBC1D32	rs9317652	PCDH9	2.717E-09	1.44E-07
rs17653341	SH3TC2	rs6507759	MIR4527HG	3.133E-09	1.56E-07
rs17083190	TBC1D32	rs978268	FGF14	3.502E-09	1.61E-07
rs10210979	ESPNL	rs12477083	HS6ST1	3.608E-09	1.61E-07
rs10210979	ESPNL	rs6936115	AL606923.2	4.387E-09	1.86E-07
rs9814172	MAGI1	rs6936115	AL606923.2	6.287E-09	2.54E-07
rs11920836	LINC01208	rs6445245	PTPRG	8.55E-09	3.30E-07
rs9311738	FHIT	rs6445245	PTPRG	1.216E-08	4.48E-07
rs10210979	ESPNL	rs11920836	LINC01208	1.283E-08	4.53E-07
rs10210979	ESPNL	rs11073328	FAM98B	1.449E-08	4.85E-07
rs9311738	FHIT	rs9807842	ZNF577	1.486E-08	4.85E-07
rs10210979	ESPNL	rs6445245	PTPRG	1.566E-08	4.92E-07
rs11920836	LINC01208	rs1446540	LINC00276	1.935E-08	5.86E-07
rs12464623	C2CD6	rs6445245	PTPRG	2.036E-08	5.95E-07
rs2067477	CHRM1	rs11920836	LINC01208	2.463E-08	6.96E-07
rs7694687	HTT	rs4507859	PAX5	3.037E-08	8.31E-07
rs204995	PBX2	rs11244	HLA-DOB	3.244E-08	8.45E-07
rs1446540	LINC00276	rs4507859	PAX5	3.288E-08	8.45E-07
rs10992568	FGD3	rs4705038	STK32A	3.749E-08	9.28E-07
rs11073328	FAM98B	rs9807842	ZNF577	3.832E-08	9.28E-07

## Conclusion

Machine learning feature selection methods are needed to enrich for attributes involved in complex interaction network effects in high dimensional data, such as GWAS and gene expression, for case-control and quantitative trait studies. In addition to interactions, machine learning methods need to handle complicated modeling scenarios, such as controlling for potential confounders from demographic data or population structure, which is a perennial challenge for GWAS data.

In the current study of GWAS data, we applied a new feature selection technique called NPDR that uses the GLM to perform regression between nearest-neighbor pair distances projected onto predictor dimensions. NPDR detects interaction structure using local nearest-neighbor information in the full space of predictors, which may be SNPs or expression levels. Using simulated GWAS, we showed that NPDR has good power to detect functional variants in a variety of simulation scenarios including case-control data with and without imbalance, quantitative trait outcomes, main effects, and multiple pairwise epistatic effects. Similar to our previous findings (McKinney et al., 2009; Le et al., 2020), NPDR is less susceptible to the curse of dimensionality than random forest because when the total number of variants increases to 10,000, the ranking of interacting variants by random forest is consistent with a random ranking, while NPDR consistently ranks the functional variants near the top.

We demonstrated NPDR’s ability to handle covariates by including the first 10 PCs in the NPDR models for a GWAS of SLE. Previously we showed that using the covariate term in NPDR can remove genes from nearest-neighbor feature selection that are confounded by sex (Le et al., 2020). In the current study, we constructed a candidate epistasis network for SLE from the filtered data, and found the ESPNL gene is a hub in the network with 15 of the 35 statistically significant interactions. There is no prior evidence for the role of ESPNL in autoimmunity, but it is ubiquitously expressed. Replication and functional investigation of these interactions are needed to identify mechanisms in the pathogenesis of autoimmunity. The lupus epistasis network also contains a significant interaction between HLA-DOB and PBX2, which are both located within the MHC class II region on chromosome 6. A limitation of this discovery analysis was the lack of a replication dataset. Another limitation is the need for SNP filtering in the current NPDR implementation in R for GWAS. Future implementations will take advantage of binary GWAS data formats for improved memory management.

## Data Availability Statement

Software available at: https://github.com/insilico/npdr.

## Author Contributions

MA and BM conceived of the project and wrote the initial draft. MA performed the analyses. CM and PG provided the statistical and biological expertise. All authors contributed to the article and approved the submitted version.

## Conflict of Interest

The authors declare that the research was conducted in the absence of any commercial or financial relationships that could be construed as a potential conflict of interest.
